# The 1-min animal test as a mental status screening examination in patients with diabetes

**DOI:** 10.1186/s12930-018-0043-0

**Published:** 2018-06-07

**Authors:** Shigeki Kinuhata, Yasuhiko Takemoto, Mariko Senda, Shiho Nakai, Erika Tsumura, Tatsuyuki Otoshi, Sadahiko Hiratani, Kazuo Fukumoto, Hiroki Namikawa, Yoshihiro Tochino, Mina Morimura, Taichi Shuto, Sadahiko Uchimoto

**Affiliations:** 1 Department of Medical Education and General Practice, Osaka City University Graduate School and Faculty of Medicine, 1-4-3, Asahi-machi, Abeno-ku, Osaka, 545-8585 Japan; 2 Fujiidera Municipal Hospital, Fujiidera, Japan

**Keywords:** 1-min mental status examination, 1-min animal test, Dementia, Revised Hasegawa Dementia Scale, Screening

## Abstract

**Background:**

Detecting and treating dementia at an early stage are important. Although the Revised Hasegawa Dementia Scale (HDS-R) is commonly used to detect dementia, it takes about 10 min to complete. In contrast, the 1-min animal test (OMAT) takes only 1 min to complete and may be a helpful screening test for general practitioners in deciding whether to proceed with administering further diagnostic tests such as the HDS-R. We sought to examine the relationship between the OMAT and HDS-R scores, and determine the cut-off OMAT score that balanced the sensitivity and specificity in identifying HDS-R-positive patients.

**Methods:**

A total of 122 consecutive patients with diabetes who visited the outpatient clinic at the Fujiidera Municipal Hospital were enrolled. The patients underwent the OMAT and HDS-R on the same day. Tests were conducted in a single-blinded manner. The relationship between the OMAT and HDS-R scores was examined using Spearman’s rank correlation. Receiver operating characteristic curve analysis was performed to identify the optimal cut-off score of OMAT that will determine whether to proceed with further diagnostic tests.

**Results:**

A strong positive correlation between the OMAT and HDS-R scores was observed (r = 0.70). The sensitivity and specificity of OMAT using cut-off scores of 12/13, 13/14, and 14/15 for HDS-R-positive patients were 0.87 and 0.66, 1.00 and 0.51, and 1.00 and 0.40, respectively among all the subjects. Similar results were obtained in a subgroup of subjects aged ≥ 65 years.

**Conclusions:**

A cut-off score of 13/14 on the OMAT balanced the sensitivity closest to 1.00 and allowed for the highest specificity for the HDS-R not only among all the patients, but also among just the patients aged ≥ 65 years. The OMAT may be an optimal screening test to determine whether to proceed with further diagnosis using HDS-R.

*Trial registration* UMIN UMIN000025260. This study is retrospectively registered on December 13th, 2016

## Background

It has been estimated that one-seventh of elderly people aged 65 years or greater suffer from dementia in Japan [1]. Ohara et al. have shown that diabetes is a significant risk factor for all-cause dementia [2]. In addition, the prevalence of Alzheimer’s disease (AD), one of the leading causes of dementia, is on the rise [3]. Dementia is one of the leading sources of caregiver burden and has emerged as a crucial public health, medical, economic, and social problem.

Several approaches to treat dementia including person-centered care, rehabilitation, training, or drugs, have recently become clinically available to prevent the progression of dementia. Therefore, it is important to perform screening tests for dementia at an early stage so that patients with dementia can be treated as early as possible to avert the progression of dementia-related behaviors and symptoms, and reduce medical, economic, and social burden alike. However, the early symptoms of dementia are difficult to detect for not only the patients and their families, but also for general practitioners. In addition, it takes about 10 min to complete the frequently used tests such as the Revised Hasegawa Dementia Scale (HDS-R) [4, 5] or the Mini-Mental State Examination (MMSE) [6], which is often difficult to spare in busy out-patient clinical settings.

Recently, the 1-min animal test (OMAT), which is a 1-min mental status examination using animal names measuring verbal category fluency, has been developed. Verbal fluency can be classified into category fluency and letter fluency. Previous studies have shown that category fluency tasks are better at discriminating between control subjects and patients with dementia or mild cognitive impairment, when compared with letter fluency [7–9]. Moreover, the category “animals” is more appropriate than “vegetables” to measure semantic category fluency because it is not confounded by sex-related differences [10]. Therefore, we decided to use the OMAT. Previous studies have validated the relationship between the OMAT and MMSE and demonstrated a positive correlation between OMAT and MMSE scores [7, 10, 11]. However, to the best of our knowledge, there has been no investigation into an association between the OMAT and HDS-R scores. The OMAT may be a helpful screening test for general practitioners in deciding whether to proceed with administering further diagnostic tests such as the HDS-R.

We sought to examine the relationship between the OMAT and HDS-R scores, and also the cut-off score of OMAT that balanced the sensitivity closest to 1.00 and enabled the highest specificity for the HDS-R-positive status; this would help us determine whether to proceed with administering the HDS-R in all consecutive patients with diabetes as well as in just the diabetic patients aged 65 years or older.

## Methods

A total of 122 consecutive patients with diabetes who visited the outpatient clinic at Fujiidera Municipal Hospital from January 2014 to June 2015 were enrolled in this study. A diabetologist working at the hospital administered the OMAT and HDS-R tests. Tests were conducted in a single-blinded manner on the same day. The study protocols were in accordance with the Declaration of Helsinki, and were approved by the ethics committee of the Osaka City University (number 3496). Informed consent was obtained from all the subjects or their close relatives prior to participation.

### 1-min animal test

The OMAT is a quick and simple examination for category fluency that asks subjects to list as many different animals as possible in 1 min [7, 12]. A previous report has shown that a cut-off score of 13/14 on the OMAT was able to distinguish patients with AD from control subjects with a sensitivity of 0.91 and a specificity of 0.81. The number of animal names that could be verbally generated by the subjects was measured in this study.

### Revised Hasegawa Dementia Scale

The HDS-R has been established in Japan and, like the MMSE, is used as a common diagnostic test for dementia; the HDS-R has been demonstrated to be diagnostically more accurate compared to the MMSE [13]. The HDS-R consists of 9 simple questions with a maximum score of 30 points. Subjects are asked to state their age, date, place, repeat 3 words, perform serial subtraction of 7 starting at 100, recall digits backwards, recall 3 words, recall 5 objects, and state the names of vegetables [5]. The number of correct answers was measured and converted into scores based on the HDS-R assessment scale. A score of 20/21 on the HDS-R has been shown to discriminate between normal cognition and dementia with a sensitivity of 0.90 and a specificity of 0.82. Therefore, patients who scored less than 21 were defined as HDS-R-positive in this study.

### Relationship between OMAT and HDS-R

In this study, we enrolled all consecutive patients with diabetes who visited our hospital from January 2014 to June 2015. However, the prevalence of dementia in Japan has been reported to be 0.0476% among individuals younger than 65 years of age, and 15.75% among individuals who are 65 years or older [1]. Therefore, we examined the correlation between the OMAT and HDS-R scores, as well as the OMAT cut-off score to determine whether to proceed with administering the HDS-R, not only among all the patients but also in a subgroup of patients aged 65 years or greater.

### Statistical analysis

The Spearman’s rank correlation coefficient was used to determine the correlation between the OMAT and HDS-R scores. The receiver operating characteristics (ROC) curve analysis was used to determine the OMAT cut-off score that could identify HDS-R-positive patients [14]. The cut-off score was defined as that balancing the sensitivity closest to 1.00 while enabling the highest specificity on the ROC curve in order for the OMAT to be an optimal screening test in deciding whether to proceed to administering the HDS-R. Results with p values less than 0.05 were considered statistically significant. The statistical analyses were performed using IBM SPSS 22.0 (Chicago, IL, USA).

## Results

The baseline characteristics of the 122 subjects used for the analysis are shown in Table 1. We enrolled 122 consecutive patients with diabetes who visited the outpatient clinic at the Fujiidera Municipal Hospital in this study. The age range of the study subjects was 40–89 years. The analyses were repeated in a subgroup of patients who were 65 years or older (n = 93; median age 75 years; interquartile range 70–80 years).

**Table 1:** Baseline characteristics of the study sample (N = 122)

Variable	
Men/women, n/n	63/59
Age (years)^a^	72 (65–78)
1-min animal test score^a^	13 (10–16)
Revised Hasegawa’s dementia scale score^a^	25 (20–28)

*N* total study sample size, *n* number of males/females in group

^a^Values are expressed as median (interquartile range)

**Fig. 1:**
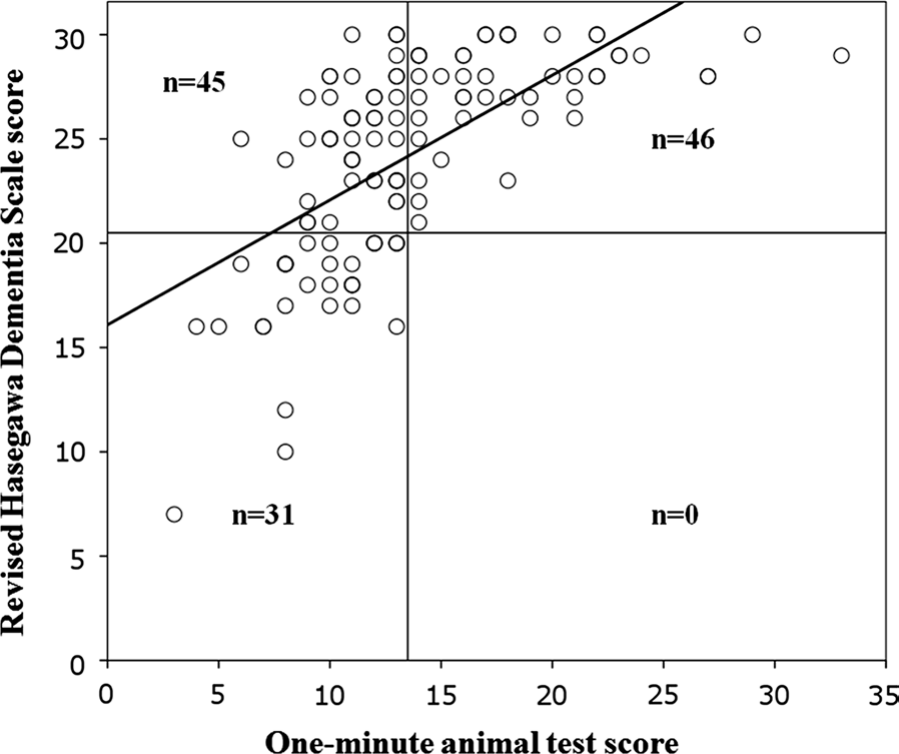
Relationship between the 1-min animal test and Revised Hasegawa Dementia Scale scores. n = 31, the number of patients with the 1-min animal test score < 14 and Revised Hasegawa’s dementia scale score < 21. n = 0, the number of patients with the 1-min animal test score ≥ 14 and Revised Hasegawa’s dementia scale score < 21. n = 45, the number of patients with the 1-min animal test score < 14 and Revised Hasegawa’s dementia scale score ≥ 21. n = 46, the number of patients with the 1-min animal test score ≥ 14 and Revised Hasegawa’s dementia scale score ≥ 21. r = 0.70 (Spearman’s rank correlation), p < 0.01

Figure 1 displays the relationship between the OMAT and HDS-R scores. A strong positive correlation was observed between the OMAT and HDS-R scores (r = 0.70, p < 0.01).

**Fig. 2:**
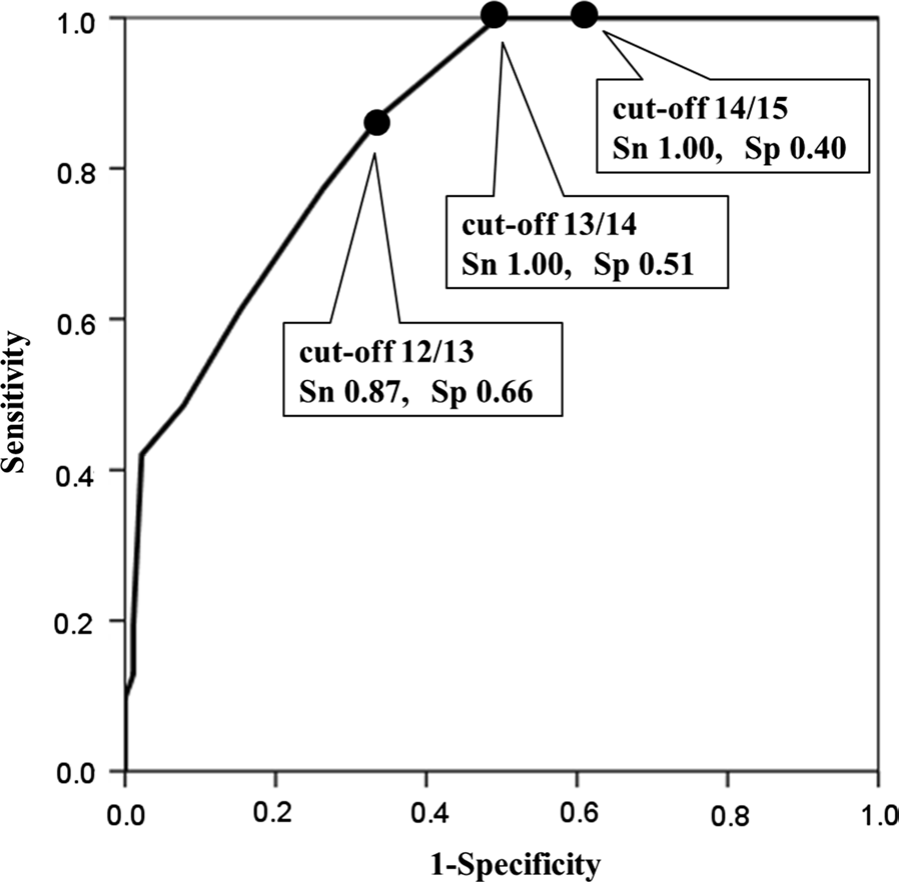
Receiver operating characteristic curve analysis. The receiver operating characteristic curve analysis of the 1-min animal test for the cut-off score of 20/21 on the Revised Hasegawa’s Dementia Scale

Figure 2 displays the results of the ROC curve analysis to determine the optimal OMAT cut-off score that can identify HDS-R-positive individuals. The OMAT scores of 12/13, 13/14, and 14/15 identified HDS-R-positive patients with sensitivities of 0.87 (0.70–0.96; 95% CI, confidence intervals), 1.00 (0.84–1.00; 95% CI), and 1.00 (0.84–1.00; 95% CI), and with specificities of 0.66 (0.55–0.76; 95% CI), 0.51 (0.40–0.61; 95% CI), and 0.40 (0.30–0.50; 95% CI), respectively. Hence, the OMAT score of 13/14 was able to balance the sensitivity closest to 1.00 while enabling the highest specificity in identifying HDS-R-positive patients.

A strong positive correlation was observed between the OMAT and HDS-R scores (r = 0.62; p < 0.01) in the subgroup analysis as well. The ROC curve analysis showed that the OMAT scores of 12/13, 13/14, and 14/15 were able to identify HDS-R-positive patients with sensitivities of 0.87 (0.69–0.96; 95% CI), 1.00 (0.83–1.00; 95% CI), and 1.00 (0.83–1.00; 95% CI), and with specificities of 0.56 (0.43–0.68; 95% CI), 0.35 (0.23–0.48; 95% CI), and 0.25 (0.15–0.38; 95% CI), respectively. Therefore, an OMAT score of 13/14 balanced the sensitivity closest to 1.00 while enabling the highest specificity in identifying HDS-R-positive patients among subjects who were 65 years or older.

## Discussion

In the present study, a strong positive correlation was observed between the OMAT and HDS-R scores among all the enrolled patients with diabetes between 40 and 89 years of age. In addition, the OMAT cut-off score of 13/14 balanced the sensitivity closest to 1.00 while enabling the highest specificity in identifying HDS-R-positive patients to determine whether to proceed with administering the HDS-R among all the patients as well as in the subgroup of patients who were 65 years or older.

To our knowledge, ours is the first study to evaluate the association between OMAT and HDS-R and show a positive correlation between their scores.

Although the MMSE has been commonly used as a screening test for dementia all over the world, it has several disadvantages. The assessment of dementia status by MMSE is prone to be influenced by the educational level of the subjects [15]. Executive functions are not fully represented in the MMSE. In addition, a previous study has shown that the HDS-R had a higher area under the ROC curve than the MMSE while identifying patients with AD [13]. Therefore, it would be useful to apply HDS-R as a standard examination in the present study.

Though HDS-R has been established as a common diagnostic test for dementia, it consists of 9 questions, takes approximately 10 min to complete, and is occasionally difficult to implement in busy out-patient clinical settings. Meanwhile, OMAT consists of one simple question and is executed in 1 min. We found that the OMAT cut-off score of 13/14 showed a sensitivity of 1.00 for identifying HDS-R-positive patients in this study. This indicates that all the patients with a score of 14 or more on the OMAT scored 21 or higher on the HDS-R, and that the HDS-R was negative for dementia when the OMAT was negative for dementia. Therefore, the OMAT can be considered as an optimal screening test to determine whether to proceed with the administration of further diagnostic tests, such as the HDS-R. In addition, a score of 13/14 was the common cut-off score to identify HDS-R-positive patients between both age groups tested, and thus may be a widely applicable index.

There have been no previous studies comparing the difference between implementing the OMAT in Japanese and any other language. The OMAT requires subjects to list as many different animals as possible in 1 min; therefore, we speculate that any differences are unlikely. Further investigations are needed to confirm this.

The present study has some limitations. First, the possibility of dementia was considered based on the HDS-R score alone, and no other tests were administered to validate this assumption. The gold standards for the diagnosis of dementia are the ICD-10, NIA-AA, and DSM-5 tests. Second, the HDS-R is an examination to assess the presence or absence of cognitive dysfunction, in particular, memory. It does not to distinguish between the different types of dementia. The OMAT is a simple examination to assess the presence or absence of cognitive dysfunction. We did not examine the association between the different types of dementia and the results of the HDS-R or the OMAT in this study. To the best of our knowledge, no previous studies have examined these associations. Further studies are necessary to address this issue. Third, the subjects of the present study were all Japanese patients with diabetes from a single hospital. Further studies involving non-diabetic patients or subjects of other ethnicities are required to improve the validity of our results. Fourth, the tests were conducted in a single-blinded manner. Further examinations using a double-blinded and independent study design are warranted to confirm our findings.

## Conclusions

The present study showed a strong positive correlation between the OMAT and HDS-R scores. The OMAT cut-off score of 13/14 balanced the sensitivity closest to 1.00 while enabling the highest specificity in the identification of HDS-R-positive patients. The OMAT may be used as an optimal screening test while deciding whether to proceed with administering further diagnostic tests such as the HDS-R not only among all the patients enrolled in the study, but also in just the subgroup of patients aged 65 years or older.
